# An Investigation of the Differences and Similarities between Generated Small-World Networks for Right- and Left-Hand Motor Imageries

**DOI:** 10.1038/srep36562

**Published:** 2016-11-04

**Authors:** Jiang Zhang, Yuyao Li, Huafu Chen, Jurong Ding, Zhen Yuan

**Affiliations:** 1 Department of Medical Information Engineering, School of Electrical Engineering and Information, Sichuan University, Chengdu 610065, China; 2 School of Information Science and Technology, Southwest Jiaotong University, Chengdu 610031, China; 3 School of Life Science and Technology, University of Electronic Science and Technology of China, Chengdu 610054, China; 4 Institute of Automation and Electronic Information, Sichuan University of Science and Engineering, Zigong 643000, China; 5 Bioimaging Core, Faculty of Health Sciences, University of Macau, Macau SAR China

## Abstract

In this study, small-world network analysis was performed to identify the similarities and differences between functional brain networks for right- and left-hand motor imageries (MIs). First, Pearson correlation coefficients among the nodes within the functional brain networks from healthy subjects were calculated. Then, small-world network indicators, including the clustering coefficient, the average path length, the global efficiency, the local efficiency, the average node degree, and the small-world index, were generated for the functional brain networks during both right- and left-hand MIs. We identified large differences in the small-world network indicators between the functional networks during MI and in the random networks. More importantly, the functional brain networks underlying the right- and left-hand MIs exhibited similar small-world properties in terms of the clustering coefficient, the average path length, the global efficiency, and the local efficiency. By contrast, the right- and left-hand MI brain networks showed differences in small-world characteristics, including indicators such as the average node degree and the small-world index. Interestingly, our findings also suggested that the differences in the activity intensity and range, the average node degree, and the small-world index of brain networks between the right- and left-hand MIs were associated with the asymmetry of brain functions.

Motor imagery (MI), as a typical mental representation of motor movements^1^,^2^,^3^,^4^, plays an essential role in the fields of athletic rehabilitation, skills training, and brain-computer interfaces (BCIs)^4^,^5^,^6^. Previous work has validated that the intensities and volumes of brain cortical activation are different between the right- and left-hand MIs, which is characterized as asymmetry and lateralization^1^,^7^. However, the brain activation patterns rely heavily on interaction and coordination among multiple cortical regions^8^,^9^. Therefore, an investigation of the interactions and differences of the brain activity networks underlying right- and left-hand MIs can provide new approaches toward an improved understanding of the neural mechanism of MI.

Complex network theory has been developing into a robust analysis tool for capturing the features of brain networks. Complex networks enable the exploration of the topological relationships of nodes and edges but exhibit the characteristics of small-world networks and are scale free^10^,^11^. Interestingly, complex network analysis has been performed in many fields^12^,^13^,^14^ in which small-world or scale-free properties have been identified for many real networks^12^,^13^,^14^,^15^. More specifically, recent work on brain connectivity revealed that functional networks underlying cognition or neurological and psychiatric disorders also have small-world statistical properties^12^,^15^,^16^. Consequently, in this study, it is hypothesized that large-scale functional brain networks during right- and left-hand MIs also have small-world properties. If that is the case, what type of differences and similarities are expected between right- and left-hand MIs using small-world measurements? To address this question, a systematic investigation was performed using fMRI measurements to analyze the complex functional brain networks of different single-hand MIs based on the small-world network theory.

The novel results from the present work indicated that there were differences in the indicators of small-world properties between the functional brain networks of MI and the random networks. Our findings also suggested that the brain networks for the right- and left-hand MIs exhibited very similar small-world properties, such as the clustering coefficient, the average path length, the global efficiency, and the local efficiency. Meanwhile, we also discovered that the constructed small-world brain networks indeed manifested differences between the right- and left-hand MIs, including indicators such as the average node degree and the small-world index. The small-world indicators express the feature information of the neural network mechanism^17^,^18^,^19^. It is anticipated that the investigation into differences and similarities of the indicators of small-world brain networks during right- and left-hand MIs will contribute to improving the understanding of the neural mechanism of MI.

## Materials and Methods

### Subjects

Twelve right-handed healthy subjects (aged 20–24 years, five females and seven males) participated in the fMRI study. All the recruited subjects had no history of neurological or psychiatric disorders. Handedness was assessed by the Edinburgh Handedness Inventory (EHI), and the average handedness score was 90.7, with a standard deviation of 6.4. All the subjects were required to sign the informed consent documents before the experimental tests. The protocol for the clinical trial was approved by the Institutional Review Board at the West China Hospital of Sichuan University and was carried out in accordance with the relevant guidelines, including any relevant details.

### Tasks

The fMRI data were acquired in two sessions: one for the left-hand MI and motor execution (ME) and the other for the right-hand MI and ME. Each session consisted of 10 runs, and the time duration for each single run was 30 s (4 s for sequence informing, 10 s for MI, 6 for ME, and then 10 s for rest). The task started with 4 s of sequence informing, in which the picture cues were presented on the PC screen to display a random order of finger tapping (for instance, ring-index-little-middle or index-middle-ring-little). Subsequently, the subjects were required to imagine tapping their fingers in the order defined by the visual stimuli on the screen. The stimuli for the MI lasted 10 s, during which the screen was black. After the MI, another cue was presented on the screen, requesting that the subjects perform the finger-tapping task during the duration of 6 s, which was exactly the same as they had imagined. At the end, the subjects were instructed to take 10 s of rest, during which the screen turned black again. The MI task was followed by a ME task to ensure that the subjects concentrated on the MI and imagined the finger tapping correctly^7^. In this study, our aim was to use the small-world network analysis to explore the neural mechanism of the MI rather than the ME. Consequently, for the paradigm design, to ensure that we could acquire high-quality MI data, the duration the MI task for each trial was longer than that of the ME. Note that all participants were requested to receive one hour of training on how to perform the tasks during the experiments before undergoing the fMRI scanning. At the end of the scanning session, the subjects were asked to write a statement on their performances during the completion of the tasks. All subjects reported having excellent performances for both the ME and MI tasks.

### Data acquisition

Data acquisition was performed using a 3T GE-Signa scanner (Huaxi MR Research Center, Chengdu, China). The gradient-recalled echo planar imaging (EPI) sequence was utilized with the following parameters: 30 transverse slices, TR = 2s, TE = 30 ms, FOV = 24 cm, matrix = 64 × 64, slice thickness = 5 mm (without gap), voxel size = 3.75 mm × 3.75 mm × 5 mm, and flip angle = 90°. The total scanning time was 310 s, and 155 images were acquired in total.

### Image pre-processing

Before data analysis was performed, SPM8 software (The Wellcome Department of Imaging Neuroscience Institute of Neurology, University College London, London; http://www.fil.ion.ucl.ac.uk) and DPARSF (http://rfmri.org/DPARSF) SPM-based toolboxes were used to prepare the images. The first five images were eliminated from each session to allow for magnetization equilibrium and for the subjects to get adapt to the circumstances^7^. The remaining images were further preprocessed according to four steps: (1) correcting differences in the image acquisition time between different slices; (2) realigning a time-series of images to remove movement artifacts; (3) normalizing the images to the standard SPM8 EPI template (the normalization step warps each individual subject into the standard space with a resolution of 3 mm × 3 mm × 3 mm based on the Montreal Neurological Institute (MNI) template); and (4) smoothing the images, with the full width at half-maximum (FWHM) specified as 8 mm. The low-frequency noise in the fMRI signal was filtered using a high-pass filter with a cutoff frequency of 1/128 Hz.

### Identification of activated brain regions

The *T*-test (*p* < 0.001) in SPM8 was used to detect brain activation regions during MI based on the contrast between the MI and the rest conditions. The fMRI results were generated from group data analysis, in which the threshold of the activation maps was set as *p* < 0.001 (*t  *>4.587) and the degree of freedom d*f* = 10.

### Matrix of correlation coefficients among different brain regions

The functional brain images of each subject were mapped to the automated anatomical labeling (AAL) brain template and then separated into ninety anatomical regions of interest (ROIs) within the cortex and subcortex (excluding cerebellum)^20^. The time series of all voxels from each brain region were extracted and averaged, and thus the average blood oxygenation level-dependent (BOLD) signal of each brain region was generated. Band-pass filters (0.01–0.08 Hz) were implemented for the BOLD signals to reduce both the low-frequency drift and the high-frequency noise^21^,^22^,^23^. Moreover, various sources of interference, including head motion parameters, white matter signals, cerebrospinal fluid signals, and global mean signals, were regressed out, and the influences from the ME and visual tasks were also removed. For the brain network analysis, Pearson’s linear correlation coefficients among the time series from ninety anatomical ROIs were calculated. We further binarized the Pearson correlation coefficient matrix, and these results demonstrated that the network topological properties were correlated with the thresholds of correlation coefficients. Recently, several methods have been developed to show how to select the range of thresholds based on the significances of correlation coefficients and the connectivity of brain networks (i.e., *K*_*net*_ ≥ 2ln(90) ≈ 9)^22^,^24^. The same strategies were adopted for the present study to quantify a series of thresholds (*T*) of correlation coefficients (0.225 ≤ *T* ≤ 0.550) to detect network topological properties. When the correlation coefficients were binarized, the smaller absolute values of correlation coefficients were considered non-significant connections, which should be ruled out by the threshold before the network analysis was implemented. Specifically, the brain regions were considered as the nodes of the brain network and the brain connections (correlation coefficients) as the edges of the network. Meanwhile, the connection of a node itself was set to 0 and the value of edge to 0 if the absolute value of the correlation coefficient was less than the threshold; otherwise, the edges were set to 1. Then, the measures of brain networks were further processed as described below to characterize the properties of the small-world networks.

### Small-world brain network analysis

Specific indicators such as the clustering coefficient of the network, the average path length, the global efficiency, the local efficiency, the average node degree, and the small-world index were utilized here to evaluate the topological properties of small-world networks. In particular, the clustering coefficient *C*_*net*_ of the network is defined as^10^,^25^


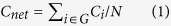


in which *C*_*i*_ is the clustering coefficient of node *i*, *G* is the set of whole nodes within the network, and *N* is the number of nodes. The clustering coefficient of node *i* is written as


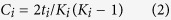


in which *t*_*i*_ is the number of edges in the subgraph *G*_*i*_, which is defined as the graph including the nodes that are associated with the *i*th node, i.e., directly connected to the *i*th node with an edge (excluding node *i*)^24^. In addition, *K*_*i*_ is the number of nodes directly connected to the node *i* and is defined as the degree of node *i*^10^,^25^. In particular, the average path length of network is defined as^10^,^25^


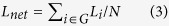


in which *L*_*i*_ is the average shortest path of node *i*, 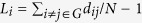, where *d*_*ij*_ is the shortest path between the pair of nodes *i* and *j*. The global efficiency of the network is denoted as^25^,^26^


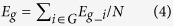


in which *E*_*g*___*i*_ is the efficiency of node *i*,


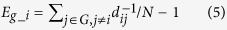


The local efficiency of the network is written^25^,^26^,


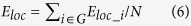


in which *E*_*loc*___*i*_ is the local efficiency of node *i*,


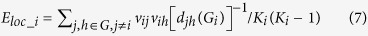


where *v*_*ij*_ = 1 when link (*i*, *j*) exists (when *i* and *j* are neighbors); *v*_*ij*_ = 0 otherwise. Further, *d*_*jh*_(*G*_*i*_) is the length of the shortest path between the pairs of nodes *j* and *h*, which contains only neighbors of node *i*. Finally, the average node degree of network is defined as^25^


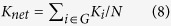


When the clustering coefficient of the brain functional networks *C*_*net*_ and the average path length *L*_*net*_ were calculated based on the above equations for the functional networks, the random networks corresponding to brain functional networks were constructed so that their small-world properties could be compared with those of the brain networks during the right and left-hand MIs. To make the random networks better match their related functional networks, in this study, we formulated one hundred random networks for the right- and left-hand MIs using the Markov chain algorithm^24^,^26^,^27^,^28^. For this algorithm, a pair of edges (links) of networks is randomly selected, for instance, an edge (link) between nodes *i*_1_ and *j*_1_ and another edge between nodes *i*_2_ and *j*_2_. The two edges are then rewired in such a way that simultaneously combines *i*_1_ with *j*_2_ and *i*_2_ with *j*_1_. However, if one or both of these new edges already exist in the network, this step is aborted, and a new pair of edges can be selected^24^,^28^. This procedure is repeated until the topological structure of the original matrix is randomized, resulting in a random graph. Here, the node degrees of random networks should match those of the brain functional networks^21^,^24^. Then, the mean of clustering coefficients and the mean of average path lengths from the one hundred random networks were specified as the clustering coefficient *C*_*random*_ and the average path length *L*_*random*_ of the random networks, respectively^24^. If we define 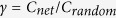 and 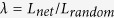, the small-world index can be determined. This index should be larger than 1.0 for small-world networks^25^,^29^ and was expressed as


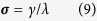


## Results and Discussion

**Figure 1:**
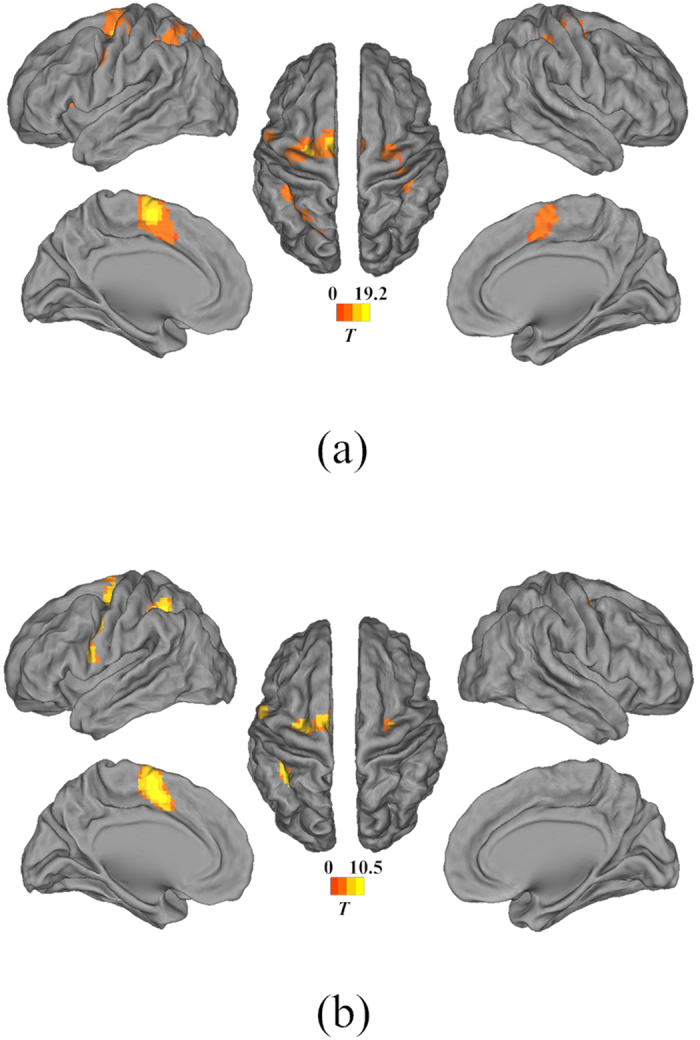
Activation maps for the different single-hand MI tasks. The statistical parametric maps of group data thresholded at *p* < 0.001 (*T* > 4.587) and the degree of freedom d*f* = 10. (**a**) The activated regions during the left-hand MI. (**b**) The activated regions during the right-hand MI.

**Figure 2:**
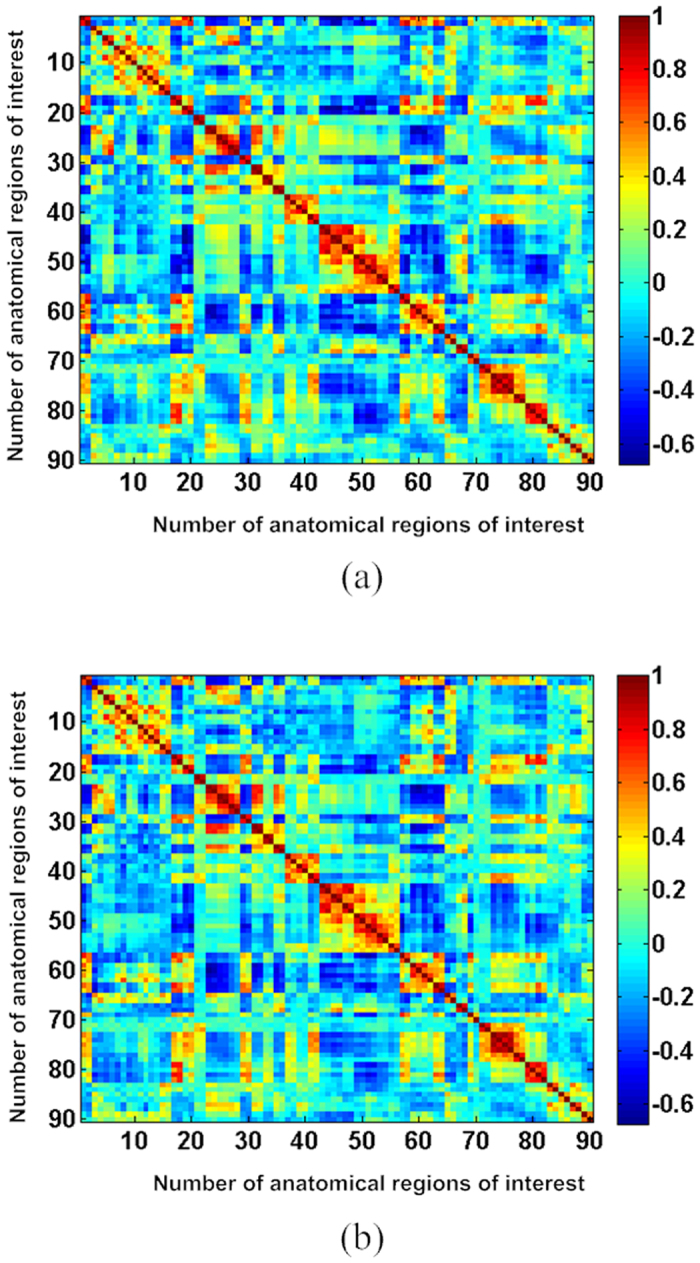
Matrixes of correlation coefficients among the different brain regions: (**a**) the left-hand MI; (**b**) the right-hand MI. The horizontal and vertical axes denote the numbers of anatomical regions of interest, in which the numbering sequence of ROIs is consistent with that of anatomical regions of interest in the AAL template. The color scale indicates the values of the correlation coefficients.

Figure 1 shows the brain activation regions that were identified by using the group data analysis of the subjects during single-hand MI tasks (the datasets of one subject were discarded because of head movements). The brain images were constructed using the threshold value when *p* < 0.001 (*t* > 4.587) and the degree of freedom d*f* = 10 based on the *t*-statistics in SPM8. Specifically, Fig. 1(a) shows the brain regions activated during the stimulus period of the left-hand MI, whereas Fig. 1(b) shows the areas activated during the right-hand MI. The colored areas in Fig. 1 represent the activated clusters within the brain cortex. Detailed information regarding the activated cluster sizes, the MNI coordinates of the peak values, the statistical *t-*values and locations during the completion of the left-hand and right-hand MI tasks is provided in Tables 1 and 2, respectively. It can be observed from Fig. 1 that compared with the right-hand MI, the left-hand MI showed more activated clusters in both hemispheres and larger statistical *t*-values in the peaks of clusters (Fig. 1, Tables 1 and 2). This result was largely due to the asymmetry of activity in the motor cortex from the right-handed subjects^30^,^31^,^32^,^33^,^34^,^35^,^36^,^37^. Figure 2 shows the visual matrixes of Pearson correlation coefficients for the different single-hand MI analyses. The links among brain nodes in Fig. 2 are the means of correlation coefficients of the associated nodes from the subjects. For the functional brain networks, the larger absolute values of correlation coefficients represent the stronger correlations between the nodes, whereas the smaller ones denote the weaker correlations.

**Figure 3:**
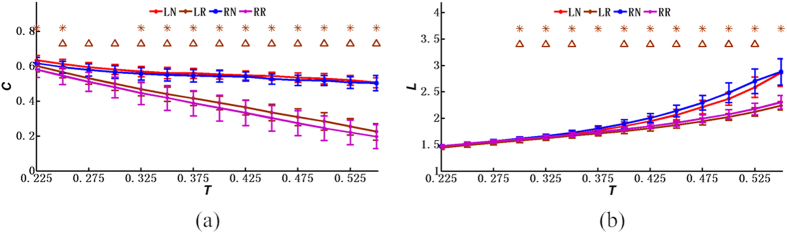
Network properties (mean ± SD) under different thresholds: (**a**) the clustering coefficient; (**b**) the average path length. LN denotes the left-hand MI, whereas RN represents the right-hand MI. LR and RR represent the random networks, respectively, with degrees matching those of the brain functional networks during the left-hand MI and right-hand MI. *p < 0.05 (*p* values of two-sample *T*-test between the left-hand MI networks and the left-hand random networks under various threshold values, Bonferroni-corrected), and ^Δ^*p* < 0.05 (*p* values of two-sample *T*-test between the right-hand MI networks and the right-hand random networks under various threshold values, Bonferroni-corrected).

**Figure 4:**
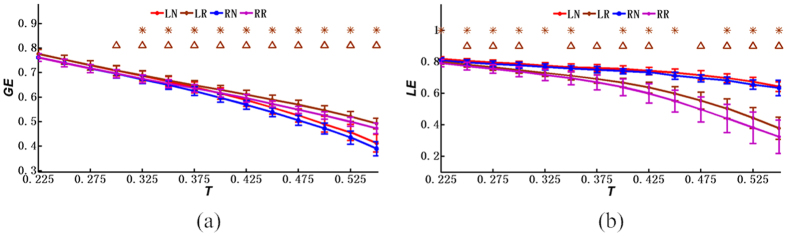
Network properties (mean ± SD) under different thresholds: (**a**) the global efficiency; (**b**) the local efficiency. LN represents the left-hand MI, whereas RN denotes the right-hand MI. LR and RR represent the random networks, respectively, with degrees matching those of the brain functional networks during the left-hand MI and right-hand MI. *p < 0.05 (*p* values of two-sample *T*-test between the left-hand MI networks and the left-hand random networks under various threshold values, Bonferroni-corrected), and ^Δ^*p* < 0.05 (*p* values of two-sample *T*-test between the right-hand MI networks and the right-hand random networks under various threshold values, Bonferroni-corrected).

**Figure 5:**
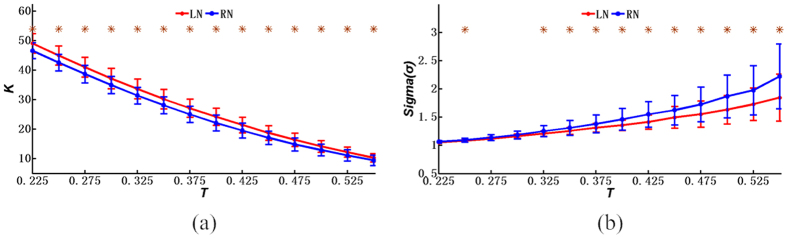
Network properties (mean ± SD) under different thresholds: (**a**) the average node degree; (**b**) the small-world index. Red represents the left-hand MI, and blue represents the right-hand MI. *p < 0.05 (*p* values of two-sample *T*-test between the left-hand MI and right-hand MI under various threshold values, Bonferroni-corrected).

Figures 3, 4 and 5 further demonstrate the similarities and differences of the network properties between different single-hand MIs. Figures 3, 4 and 5 show that the coefficients and indexes of brain networks during the MI tasks varied with different thresholds of the correlation coefficients. Consequently, a range of thresholds were generally utilized to analyze the properties of the small-world networks, including the clustering coefficient of brain networks, the average path length, the global efficiency, the local efficiency, the average node degree, and the small-world index^22^,^24^. As shown in Figs 3, 4 and 5, the curves were plotted in terms of the mean value and the standard deviation (SD) calculated from the subjects, with the LN curves representing the brain networks during left-hand MI and the RN curves representing right-hand MI. Meanwhile, the LR and RR curves represent the random networks with degrees matched to those of the brain functional networks during left-hand MI and right-hand MI, respectively. Note that the horizontal axes (X axes) denote the threshold values, whereas the vertical axes (Y axes) denote the property indexes of the networks.

Figure 3(a) compares the global clustering coefficients between the functional networks and random networks using the same node degree as the functional networks during the completion of MI tasks. Larger clustering coefficients were considered to indicate higher cliquishness of the networks. Interestingly, Fig. 3(a) shows that the clustering coefficients of the functional networks were larger than those of the random networks for all the threshold values. Before the statistical test, the Box-Cox function in MATLAB was used to transform the datasets from the subjects to a dataset with approximately normal distribution. Two-sample *T*-tests were performed, and we identified significant differences (*p* < 0.05, Bonferroni-corrected) between the MI brain networks and the random networks. Furthermore, the global clustering coefficients decreased with increased thresholds. The maximum values of the averaged global clustering coefficients were 0.618 and 0.635, whereas the minimum values of the mean global clustering coefficients were 0.504 and 0.507, for the right- and left-hand MI, respectively. Similarly, the computed maximum values of the averaged global clustering coefficients were 0.582 and 0.603, whereas the minimum values of the mean global clustering coefficients were 0.197 and 0.225, for the right and left random networks, respectively. Figure 3(b) presents the distributions of average path length of the networks, and the path-length values of functional networks were larger than those of the random networks for both the right- and left-hand MI. Furthermore, significant differences (*p* < 0.05, Bonferroni-corrected) between the MI brain networks and the random networks were revealed when the thresholds were greater than 0.3. Figure 3(b) also shows that the average path length increased with increased thresholds. The minimum values for the mean path length of the functional networks were 1.476 and 1.448 for the right- and left-hand MI, respectively, whereas the maximum values were 2.881 and 2.865, respectively.

The global efficiency and the local efficiency measure the information transmission capabilities of the network at the global and local levels, respectively^25^,^26^,^38^. The global efficiencies and the local efficiencies were computed and compared between the functional networks and random networks (with the same node degree as the functional networks during the completion of the MI), as shown in Fig. 4(a,b), respectively, and the statistical *T*-test results are also provided in Fig. 4. Based on Fig. 4(a), we observe that the difference in the mean values of the global efficiencies between the functional networks and the random networks was smaller than that of the local efficiencies. Furthermore, Fig. 4(b) shows that the local efficiencies of the functional networks were markedly larger than those of the random networks. Interestingly, this phenomenon appears to be a valid feature of the small-world networks^38^.

In addition, Fig. 5(a) shows the average node degree of networks for both the right- and left-hand MIs and the results of a two-sample *T*-test, through which we discovered that the degrees of networks for the left-hand MI were higher than those for the right-hand MI. The maximum values of *K*_*net*_ for the right- and left-hand MIs were 46.624 and 49.137, respectively, whereas the minimum values for these cases were 9.483 and 10.325, respectively. More importantly, it is widely recognized that a network possesses small-worldness if the small-world index σ is greater than 1^21^,^22^,^39^,^40^. Interestingly, the results shown in Fig. 5(b) indicate that both of the brain networks during the right- and left-hand MIs have the organizational characteristics of small-world networks. Furthermore, differences in small-worldness were present between the functional brain networks during the right- and left-hand MIs. Figure 5(b) shows the small-world index of the functional brain networks from MI, from which we observed significant differences (*p* < 0.05, Bonferroni-corrected) between the left-hand MI and the right-hand MIs when the thresholds were greater than 0.325 and when simultaneously using two-sample *T*-tests. More specifically, the mean values of the small-world index during the right-hand MI were larger than those during the left-hand MI. The differences between mean values became obvious when the thresholds were ranged from 0.325 to 0.55, which was confirmed by the increased distances between the blue and red curves. Furthermore, for the functional brain networks, the small-world index increased with increased threshold values. For example, the minimum values of σ were 1.064 and 1.055 for the right- and left-hand MI, respectively, whereas the maximum values were 2.222 and 1.846, respectively, as denoted by the red and blue curves. Moreover, Fig. 3 shows that there were similarities between the right- and left-hand MIs in terms of the clustering coefficients and the average path lengths of functional brain networks. As mentioned above, our finding shown in Fig. 5 also suggested that the small-world indexes of the brain networks showed significant differences between the right- and left-hand MIs.

**Figure 6:**
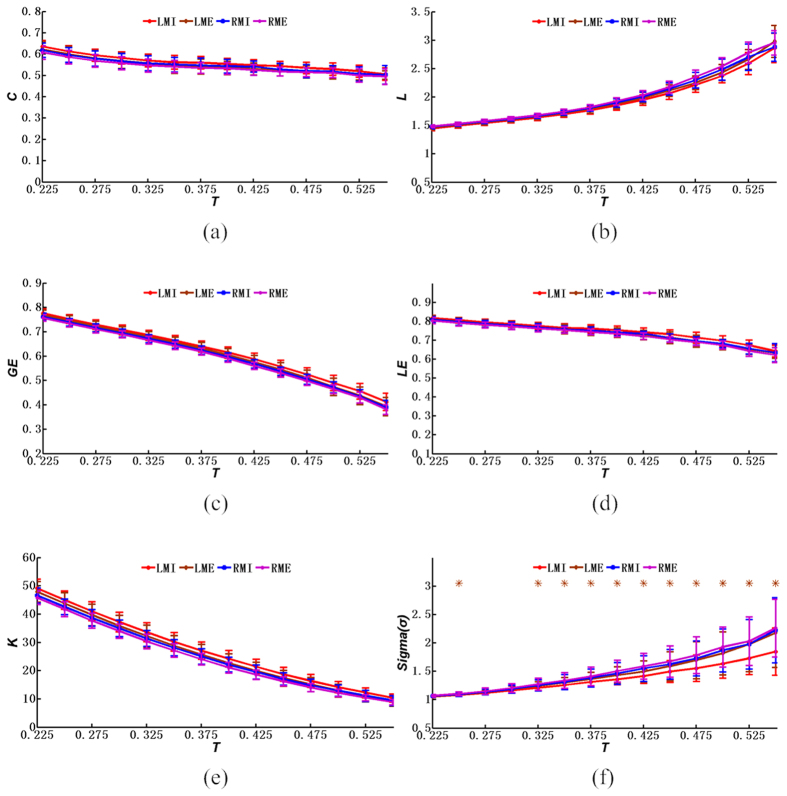
Network properties (mean ± SD) under different thresholds: (**a**) the clustering coefficient; (**b**) the average path length; (**c**) the global efficiency; (**d**) the local efficiency; (**e**) the average node degree; (**f**) the small-world index. LMI denotes the left-hand MI, whereas RMI indicates the right-hand MI. LME denotes the left-hand ME, whereas RME indicates the right-hand ME. *p < 0.05 (p values of two-sample *T*-test between the left-hand ME and left-hand MI under various threshold values, Bonferroni-corrected).

Further, we also processed the fMRI data from ME and compared the ME results with those from the MI results, although this is not the focus of this study. To allow comparisons of the differences in network properties between MI and ME, small-world analysis was also performed for ME, and the results are provided in Fig. 6. Figure 6(a) shows that the mean values of the clustering coefficient of brain networks of ME are similar to that of MI. Similarly, as shown in Fig. 6(a–e), this result is also the case for other small-world properties of the functional brain networks between the ME and the MI, such as the mean values of the average path length, the global efficiency, the local efficiency, and the average node degrees. However, as shown in Fig. 6(f), the small-world properties in the networks of left-hand MI and left-hand ME did exhibit clear differences according to the mean values of the small-world index. The results of this analysis showed that the small-world index of the brain networks during left-hand MI is significantly different from those of the ME and the right-hand MI. In particular, the two-sample *T*-test was performed based on the values of the small-world index between the brain networks of ME and MI. This result showed that the small-world index of the brain networks during left-hand MI is clearly different from those during the ME and the right-hand MI. More importantly, the statistical analysis indicated that there were significant differences between the small-world indexes of the brain networks of the left-hand ME and the left-hand MI when the thresholds were between 0.225 and 0.55 after the data were transformed to produce a normal distribution. However, no significant differences were identified between the left- and right-hand MEs or between the right-hand ME and the MI.

In addition, to ensure that no overt movement is produced during the completion of the MI task, it is good to use EMG systems to monitor the neural electrical signals of the hands and fingers. However, due to safety concerns regarding our present imaging facilities, it is very challenging to fuse the EMG system with the fMRI system in our fMRI recordings. Instead, during the fMRI data recording, we used a video camera to monitor the subject’s status and to check whether they performed any kind of body movement. And the subjects were required to perform a behavioral test that followed the same procedure as the fMRI scan. At the end of the scanning session, the subjects were surveyed via a simple questionnaire to ensure that they had not fallen asleep, had not moved their head or body, and had succeeded in performing both the ME and MI tasks in response to the instructions provided. In addition, the subjects were asked to assess and categorize their ability to perform the MI task, using a simple three-level scale (i.e., “hard”, “neither easy nor hard” or “easy”); all the subjects reported that the task was “easy”. It may be considered a limitation of the present study that we did not use a more detailed index to assess the subjects’ performance of the MI task. It should be also pointed out that it is possible that MI can generate some effect on ME, for instance, MI might be considered as a practice for ME. However, MI was determined as our research aim whereas ME was not. More importantly, the MI practice was not the real ME practice and the influence of MI practice on ME task should be very weak. Consequently, for our initial paradigm design, the MI task was followed by a ME task to ensure that the subjects could concentrate on the MI and imagine the finger tapping correctly^7^. Meanwhile, MI and ME are totally different tasks and ME might not be executed differently when MI would not precede it. Indeed, if there are some bad runs existing during ME data acquisition due to the effect of MI (tapping sequences are totally in bad order), it should be excluded for further data analysis.

Previous investigations of the brain activation patterns during MI reported changes in the intensity of brain activity, revealed the lateralization and asymmetry of brain functions^1^,^7^,^33^, and also identified the brain networks^17^,^40^. However, previous studies of the properties of small-world networks have not addressed the differences or the similarities between right- and left-hand MIs. So, the aim of this study is to conduct the small-world network analysis to generate the indicators of the MI, which can be used to identify similarities and differences between left- and right-hand MIs. In the system of a complex network, the clustering coefficient of the network depicts the local or small-group efficiency in information transfer within the network^17^. By contrast, the average path length of the network describes the global efficiency and the capacity for parallel transmission of information^17^. The global and local efficiency of the network are correlated to the functional efficiency with which the system can transmit information between any two nodes via multiple parallel paths^17^. The average node degree represents the network density, in which the network connections are sparse when the average node degree is small, whereas the network connections are denser if the average node degree is larger^17^. The small-world index summarizes the small-world properties of brain functional networks^18^,^24^. As such, it is crucial to detect and compare the functional brain topological properties to better understand the neural mechanisms of the brain information networks in right- and left-hand MIs. The topological properties of small-world networks indicate that the brain has efficient organization characteristics to support different tasks^19^. In this study, our findings suggested that the brain activation intensity from the left-hand MI was stronger than that from the right-hand MI in relevant cortical areas. Furthermore, we revealed the similarities and differences in the complex networks of the brain during the right- and left-hand MI tasks based on the clustering coefficient, the average path length, the global efficiency, the local efficiency, the average node degree, and the small-world index. In particular, the differences in the brain activity intensity and range, the average node degree and the small-world index of the brain networks further confirmed the asymmetry of brain function during the right- and left-hand MI tasks.

## Additional Information

**How to cite this article**: Zhang, J. *et al*. An Investigation of the Differences and Similarities between Generated Small-World Networks for Right- and Left-Hand Motor Imageries. *Sci. Rep.* **6**, 36562; doi: 10.1038/srep36562 (2016).

**Publisher’s note:** Springer Nature remains neutral with regard to jurisdictional claims in published maps and institutional affiliations.

## Figures and Tables

**Table 1:** Activated clusters during the left-hand MI.

Cluster	Size (voxels)	Peak MNI coordinates	*t*	Location	BA
1	773	−6 −6 63	19.1999	Supp_Motor_Area_L	6/32
				Supp_Motor_Area_R	
				Frontal_Sup_L	
				Precentral_L	
2	18	−54 3 36	8.1763	Precentral_L	6
3	66	−30 −60 66	6.6869	Parietal_Sup_L	7/40
				Parietal_Inf_L	
4	15	−30 24 12	8.7395	Insula_L	48
5	10	−18 −75 60	5.1578	Parietal_Sup_L	7

**Table 2:** Activated clusters during the right-hand MI.

Cluster	Size (voxels)	Peak MNI coordinates	*t*	Location	BA
1	269	−9 −3 60	10.4856	Supp_Motor_Area_L	6/32
				Precentral_L	
				Frontal_Sup_L	
2	35	−57 6 18	7.1953	Precentral_L	6/48
				Frontal_Inf_Oper_L	
3	96	−33 −48 48	9.6673	Parietal_Inf_L	2/7/40
				Parietal_Sup_L	
4	71	−24 −6 6	6.8033	Putamen_L	34/48
				Pallidum_L	
5	17	30 −9 57	5.5445	Precentral_R	6
				Frontal_Sup_R	
